# Multi-color imaging of the bacterial nucleoid and division proteins with blue, orange, and near-infrared fluorescent proteins

**DOI:** 10.3389/fmicb.2015.00607

**Published:** 2015-06-17

**Authors:** Fabai Wu, Erwin Van Rijn, Bas G. C. Van Schie, Juan E. Keymer, Cees Dekker

**Affiliations:** Department of Bionanoscience, Kavli Institute of Nanoscience, Delft University of TechnologyDelft, Netherlands

**Keywords:** fluorescent proteins, bacterial cell division, bacterial chromosome, MinD, HU, FtsZ, TagRFP-T, TagBFP

## Abstract

Studies of the spatiotemporal protein dynamics within live bacterial cells impose a strong demand for multi-color imaging. Despite the increasingly large collection of fluorescent protein (FP) variants engineered to date, only a few of these were successfully applied in bacteria. Here, we explore the performance of recently engineered variants with the blue (TagBFP), orange (TagRFP-T, mKO2), and far-red (mKate2) spectral colors by tagging HU, LacI, MinD, and FtsZ for visualizing the nucleoid and the cell division process. We find that, these FPs outperformed previous versions in terms of brightness and photostability at their respective spectral range, both when expressed as cytosolic label and when fused to native proteins. As this indicates that their folding is sufficiently fast, these proteins thus successfully expand the applicable spectra for multi-color imaging in bacteria. A near-infrared protein (eqFP670) is found to be the most red-shifted protein applicable to bacteria so far, with brightness and photostability that are advantageous for cell-body imaging, such as in microfluidic devices. Despite the multiple advantages, we also report the alarming observation that TagBFP directly interacts with TagRFP-T, causing interference of localization patterns between their fusion proteins. Our application of diverse FPs for endogenous tagging provides guidelines for future engineering of fluorescent fusions in bacteria, specifically: (1) The performance of newly developed FPs should be quantified *in vivo* for their introduction into bacteria; (2) spectral crosstalk and inter-variant interactions between FPs should be carefully examined for multi-color imaging; and (3) successful genomic fusion to the 5^′^-end of a gene strongly depends on the translational read-through of the inserted coding sequence.

## Introduction

The use of fluorescent proteins (FPs) has greatly advanced our understanding of the subcellular architecture of bacteria. Soon after the first cloning of the green fluorescence protein (*gfp*) gene from *Aequorea victoria* (Prasher et al., 1992) and its first application as fluorescence marker *in vivo* (Chalfie et al., 1994), it was successfully adopted to visualize essential proteins involved in cell division and division-site selection in bacteria, such as FtsZ/FtsA (Ma et al., 1996) and MinE/MinD/MinC (Raskin and de Boer, 1997, 1999; Hu and Lutkenhaus, 1999). The results from these studies planted the significant notion that the intracellular environment of bacteria is not only structured, but also extremely dynamic. Other hallmarks include cytoskeletal filaments responsible for cell shape maintenance (Jones et al., 2001; Ausmees et al., 2003; Domínguez-Escobar et al., 2011; Garner et al., 2011; van Teeffelen et al., 2011), and polarly localized proteins involved in chemotaxis, virulence and metabolism (Sourjik and Berg, 2000; Charles et al., 2001; Lindner et al., 2008; Li and Young, 2012). Recently, several large fluorescent-fusion libraries have been constructed for *Escherichia coli* and *Caulobacter crescentus*, allowing genome-scale, quantitative studies of protein localization, which is especially powerful when accompanied by the development of a quantitative analysis toolbox (Kitagawa et al., 2006; Werner et al., 2009; Taniguchi et al., 2010; Kuwada et al., 2015).

Two decades of efforts have expanded the spectrum of FPs to a full range, showing strong promise for multi-color imaging. However, the application of many FPs for live cell imaging of bacteria have been hindered by various factors. Most prominently, the fast synthesis and degradation of protein in bacteria (in contrast to eukaryotes) demands fast-folding of FPs. This is clearly indicated by the fact that mutations improving the folding properties of EGFP generation (which resulted in SBFP2, SCPF3A, SGFP2, and SYFP2), enhanced their effective brightness by several folds in bacteria, while this was much less the case when expressed in mammalian cells (Kremers et al., 2006, 2007). Also, the orange FPs mOrange and mKO were not visible in live *E. coli* due to slow maturation, with the latter only visible after an overnight incubation after fixation (Alexeeva et al., 2010). The fast-folding properties of some FPs such as Venus, mCherry, and sfGFP have shown advantages for functional fusions (Osawa and Erickson, 2005; Bendezú et al., 2009; Dinh and Bernhardt, 2011), and have been broadly used. On the other hand, these fast-folding FPs do not always provide the native protein localization patterns (Landgraf et al., 2012). Furthermore, the degree of oligomerization, the brightness, the photostability, as well as the spectral separation between FPs are properties no less essential for successful capture of native events at the demanded spatial and temporal resolution. However, thus far, these factors have been barely quantified in live bacteria.

Here, we set out to expand the spectrum of FPs for live cell imaging in *E. coli* by labeling the cytosol, the nucleoid, as well as the division proteins. HU-2, encoded by *hupA* gene, is a subunit of nucleoid-associated proteins HU, which serves as a marker for the nucleoid that was previously shown to colocalize with DAPI when fused to GFP (Wery et al., 2001). Fluorescently labeled LacI has been used as an operator-repressor system to label specific genomic loci by targeting repeated *lacO* sequences (Lau et al., 2003). Combining the HU-2 and LacI labels allows us to localize a genomic locus in the context of the global nucleoid structure. For imaging division, we focus on FtsZ proteins, which polymerize to initiate a cytokinetic ring, and whose localization is regulated by the nucleoid and MinCDE proteins (Ma et al., 1996; Hu and Lutkenhaus, 1999; Raskin and de Boer, 1999; Bailey et al., 2014; Du and Lutkenhaus, 2014). To visualize the latter, we constructed a fusion gene at the endogenous *minD* locus for expressing sfGFP-MinD proteins, which oscillate between the two cell halves of the rod-shape *E. coli* and form a time-averaged concentration gradient that have a maxima at the cell poles and minimum at the mid-cell (Raskin and de Boer, 1999).

Expanding from the FPs derived from jellyfish *A. victoria*, we examine the performances of the monomeric FPs at the blue (TagBFP), orange (TagRFP-T and mKO2), far red (mKate2), and near-infrared (dimeric eqFP670) spectral colors. All FPs used in this study are listed in **Table 1**. The latter proteins are derivatives of the FPs from the sea anemone *Entacmaea quadricolor* and *Fungia concinna*, known for their brightness, photostability, and relatively fast maturation (Shaner et al., 2008; Subach et al., 2008; Shcherbo et al., 2009, 2010; Sun et al., 2009; Morozova et al., 2010). By quantitatively comparing the brightness, photostability, and spectral properties of these proteins to other proteins with the same spectral colors, we find that these proteins function well as cytosolic labels and/or C-terminal tags, and that they provide strong advantages in brightness, photostability, and spectral separation compared to other FPs that are currently in use for bacteria. Furthermore, our approach to tag the *minD* gene at its endogenous locus revealed a detrimental effect of the coding sequence on N-terminal fusions. Finally, we combined these FP tags in bacterial strains to assess their suitability for multi-color imaging. Together, Our data provide guidelines for an optimal strategy in choosing new FPs for multi-color imaging in bacteria.

**Table 1:** Properties of the fluorescent proteins (FPs) used in this study.

FP*	λ_em_ (nm)	λ_em_ (nm)	QY	EC	Brightness	Oligomerization	Reference	Codon
EBFP2	383	448	0.56	32000	18	Monomer	Ai et al. (2007)	n.a.
SBFP2	383	448	0.47	34000	16	Monomer	Kremers et al. (2007)	n.a.
TagBFP	402	457	0.63	52000	32.8	Monomer	Subach et al. (2008)	Mammal
mCerulean	433	475	0.62	43000	26.7	Monomer	Rizzo et al. (2004)	n.a.
TagCFP	458	480	0.57	37000	21	Monomer	Evrogen	Mammal
TagGFP2	483	506	0.6	56500	33.9	Monomer	Evrogen	Mammal
sfGFP	485	510	0.65	83300	54.1	Monomer	Pedelacq et al. (2006)	Bacteria
TagYFP	508	524	0.62	50000	31	Monomer	Evrogen	Mammal
mYPet	517	530	0.77	104000	80.1	Monomer	Nguyen and Daugherty (2005)	n.a.
mKO2	551	565	0.62	63800	39.6	Monomer	Lee et al. (2013)	Yeast
TagRFP-T	555	584	0.41	81000	33.2	Monomer	Shaner et al. (2008)	Mammal
mCherry	587	610	0.22	72000	15.8	Monomer	Shaner et al. (2004)	n.a.
mKate2	588	633	0.4	62500	25	Monomer	Shcherbo et al. (2009)	Mammal
eqFP670	605	670	0.06	15700	0.9	Dimer	Shcherbo et al. (2010)	Mammal
TagRFP657	611	657	0.1	34000	3.4	Monomer	Morozova et al. (2010)	Bacteria

*The properties are: λ_em_/λ_em_, wavelengths for excitation/emission maxima; QE, quantum yield; EC, extinction coefficient; brightness are calculated from QE and EC; reported oligomerization property; and codon usage for the versions used in this study.

## Materials and Methods

### Plasmid and Strain Construction

The plasmids were constructed using Gateway cloning kit (Invitrogen, catalog # 11789013, 11791019) and Infusion EcoDry kits (Clonetech, catalog # 638912), Coding sequences of *tagYFP*, *tagGFP2*, *tagRFP-T*, *ebfp2*, *tagBFP*, *hupA*, *minDE*, and *aph frt* were respectively amplified from the pTagYFP-C1, pTagGFP2-C1, pTagRFP-T, pBAD-EBFP, pTagBFP-C1, W3110 genome, W3110 genome, and pKD13, and inserted into the Gateway entry vectors through BP reactions as described in the Gateway protocol. These and previously described entry vectors in Wu et al. (2015) were then combined through Gateway LR reaction to produce destination vectors pERB006, pFWB007, pBVS32, pFWB009, pBVS36, and pBVS37. For constructing pFWM006, we amplified the backbone of pBVS3 with *P_*lac*_* and *aph*, the *hupA* fragment from pFWB006, and the *mKO2* fragment from pyomKO2, and combined these into one plasmid through a three-fragments Infusion reaction. For construction of pFWM007, we amplified the backbone of pBVS3 with *Plac* and *aph*, the *hupA* fragment from pFWB006, and the *sBFP2* fragment from pSBFP2-C1, and combined these into one plasmid through a three-fragment Infusion reaction. Plasmid pFWZ7 was constructed through an ligation reaction (Infusion kit) with four PCR-amplified fragments, which were the backbone of pFB174 with arabinose promoter and chloramphenicol resistance gene, an *ftsZ* (5′ sequence, 1–999 bp) fragment with an 18 base overhang coding the flexible linker GSGSGS, a GGSGSS flexible linker plus *ftsZ* (3′ sequence, 991–1052 bp) plus *aph frt* sequence amplified from strain FW1370, two synthesized oligos containing the tetracysteine (TC) peptide coding sequence and the two flanking flexible linkers. The TC coding sequence was then replaced by *tagRFP-T*, *sfGFP*, and *tagCFP* to produce plasmid pFWZ4, pFWZ5, and pFWZ6 through two-fragment Infusion reactions. pFWZ0 was constructed through Gibson assembly of pFB174 backbone and the *ftsZ::aph frt* sequence amplified from strain FW1370. All plasmids are listed in **Table 2**.

**Table 2:** Plasmids used in this study.

Plasmids	Descriptions	Reference
pKD13	*aph frt (Amp^*R*^)*	Datsenko and Wanner (2000)
pKD46	*P_*ara*_::gam bet exo (Amp^*R*^)*	Datsenko and Wanner (2000)
pCP20	*Pr-flp (Amp^*R*^ Cm^*R*^)*	Datsenko and Wanner (2000)
pDonR P4-P1R	Gateway plasmid entry 1	Invitrogen
pDonR 211	Gateway plasmid entry 2	Invitrogen
pDonR P2R-P3	Gateway plasmid entry 3	Invitrogen
pDEST R4-R3	Gateway destination vector	Invitrogen
pTagBFP-C1	*P_*cmv*_::TagBFP (Kan^*R*^)*	Evrogen
pTagCFP-C1	*P_*cmv*_::TagCFP (Kan^*R*^)*	Evrogen
pTagYFP-C1	*P_*cmv*_::TagYFP (Kan^*R*^)*	Evrogen
pTagGFP2-C1	*P_*cmv*_::TagGFP2 (Kan^*R*^)*	Evrogen
pmKate2-C1	*P_*cmv*_::mKate2 (Kan^*R*^)*	Evrogen
pTagRFP-T	*P_*T7*_::tagRFP-T (Amp^*R*^)*	Shaner et al. (2008)
pNirFP-N1	*P_*cmv*_::eqFP670 (Kan^*R*^)*	Evrogen
pBAD-EBFP2	*P_*ara*_::ebfp2-6xhis (Amp^*R*^)*	Addgene, Michael Davidson
pSBFP2-C1	*P_*cmv*_::sbfp2 (Amp^*R*^)*	Kremers et al. (2007)
pyomKO2	*pFA6a-link-yomKO2 (Kan^*R*^)*	Lee et al. (2013)
pFX40	*P_*lac*_::yfp-minD minE (Amp^*R*^)*	Shih et al. (2002)
pKD3ftsQAZ	*PftsQAZ (Amp^*R*^)*	Dai and Lutkenhaus (1991)
pFB174	*P_*ara*_::mreBCD (Cm^*R*^)*	Bendezú and de Boer (2008)
pBVS3	*P_*lac*_::yfp-minD minE::aph frt (Kan^*R*^, Amp^*R*^)*	Wu et al. (2015)
pBVS4	*P_*lac*_::sfgfp-minD minE::aph frt (Kan^*R*^, Amp^*R*^)*	Wu et al. (2015)
pERA001	Gateway destination vector with *P_rha_* (*Tet^*R*^)*	This work
pERB006	*pERA001::hupA-ebfp2::aph frt (Kan^*R*^, Amp^*R*^)*	This work
pFWM007	*P_*lac*_::hupA-sbfp2::aph frt (Kan^*R*^, Amp^*R*^)*	This work
pFWB006	*pDEST::hupA-tagBFP::aph frt (Kan^*R*^, Amp^*R*^)*	This work
pBVS32	*pDEST::leuB’-Pj23100::tagRFP-T::aph frt-leuB” (Kan^*R*^, Amp^*R*^)*	This work
pEcTagRFP657	*E. coli codon-optimized TagRFP657 gene*	This work
pERB004	*pERA001::leuB’-Pj23100::tagRFP657::aph frt-leuB” (Kan^*R*^, Tet^*R*^)*	This work
pERB005	*pERA001::Pt7::tagRFP657::aph frt (Kan^*R*^,Tet^*R*^)*	This work
pFWB009	*pDEST::hupA-tagRFP-T::aph frt (Kan^*R*^, Amp^*R*^)*	This work
pFWM006	*P_*lac*_::hupA-mKO2::aph frt (Kan^*R*^, Amp^*R*^)*	This work
pFWB019	*pDEST::hupA-mKate2::aph frt (Kan^*R*^, Amp^*R*^)*	This work
pBVS36	*pDEST:: TagYFP-MinDE::aph frt (Kan^*R*^, Amp^*R*^)*	This work
pBVS37	*pDEST:: TagGFP2-MinDE::aph frt (Kan^*R*^, Amp^*R*^)*	This work
pFWZ0	*P_*ara*_::ftsZ::aph frt (Kan^*R*^, Cm^*R*^)*	This work
pFWZ4	*P_*ara*_::ftsZswtagRFP-T::aph frt (Kan^*R*^, Cm^*R*^)*	This work
pFWZ5	*P_*ara*_::ftsZswsfGFP::aph frt (Kan^*R*^, Cm^*R*^)*	This work
pFWZ6	*P_*ara*_::ftsZswtagCFP::aph frt (Kan^*R*^, Cm^*R*^)*	This work
pFWZ7	*P_*ara*_::ftsZswTC::aph frt (Kan^*R*^, Cm^*R*^)*	This work

The genomic insertions were constructed using λ Red recombination (Datsenko and Wanner, 2000) and shuﬄed between strains using P1 transduction as described previously (Wu et al., 2015). The PCR fragments from plasmids pERB006, pFWM007, pFWB006, pBVS32, pFW009, pFWM006, pBVS3, and pBVS4 were electroporated into the electro-competent cells of W3110 containing pKD46, to result in strains FW1722, FW1951 and FW1344, FW1401, FW1464, FW2455, FW1462, FW1534. The PCR fragments of pFWM007, pFWM006, and pFWB019 were amplified to replace the *mCherry* in strain RRL189 through λ Red recombination to result in strains FW1965, FW2417, and FW2450. The PCR fragments of pBVS3, pBVS36, and pBVS37 were used to replace the *ΔminD minE::cat sacB* in strain FW1363 through Red recombination to result in strains FW1480, FW1248, FW1393. Note that in strain FW1363 the *minC* gene is intact. The above listed strains were cured of kanamycin resistance using pCP20 as described in Datsenko and Wanner (2000), and listed in **Table 2**. To confirm the functionality of YFP-MinD, *Plac::yfp-minDE* was transduced from FW1462 into FW1363 to yield strain FW1463. For multi-color imaging, *hupA-tagBFP* from FW1344 was transduced into strain FW1554 to yield 1561; *ΔleuB::eqFP670* was transduced from FW1489 into FW1554 to yield FW1559; *ΔleuB::tagRFP-T* was transduced from FW1401 into FW1359 to yield FW1406; *hupA-mYPet* from FW1551 was transduced into a strain with *aph* cured from FW1965 to yield FW2480; *Plac::yfp-minDE* was transduced from FW1462 into FW1459 to yield strain FW1503; pFWZ4 was transformed into strain FW1559. All strains used are listed in **Table 3**.

**Table 3:** Bacterial strains used in this study.

Strains	Descriptions	Reference
W3110	F-, lambda-, IN(rrnD-rrnE)1, rph-1	Hayashi et al. (2006)
FW1363	W3110, *ΔminD minE::sacB cat*	Wu et al. (2015)
FW1247	W3110, *ΔleuB::Pj23100 tagBFP::aph frt*	Wu et al. (2015)
FW1268	W3110, *ΔleuB::Pj23100 tagBFP::frt*	This work
FW1722	W3110, *hupA-ebfp2::aph frt*	This work
FW2486	W3110, *hupA-ebfp2::frt*	This work
FW1951	W3110, *hupA-sbfp2::aph frt*	This work
FW2485	W3110, *hupA-sbfp2::frt*	This work
FW1344	W3110, *hupA-tagBFP::aph frt*	This work
FW1359	W3110, *hupA-tagBFP::frt*	This work
FW1561	W3110, *ΔminD minE::exorbs2-sfgfp-minD minE::frt, hupA-tagbfp::aph frt*	This work
RRL189	AB1157, *ori1::lacOx240::hygR, ter3::tetOx240::accC1 ΔgalK::tetR-mCerulean::frt, ΔleuB::lacI-mCherry::frt*	Reyes-Lamothe et al. (2008)
FW1965	AB1157, *ori1::lacOx240::hygR, ter3::tetOx240::accC1 ΔgalK::tetR-mCerulean::frt, ΔleuB::lacI-sbfp2::aph frt*	This work
FW1551	W3110, *hupA-mYPet::aph frt*	This work
FW2480	AB1157, *ori1::lacOx240::hygR, ter3::tetOx240::accC1 ΔgalK::tetR-mCerulean::frt, ΔleuB::lacI-sbfp2::frt, hupA-mYpet::aph frt*	This work
FW1401	W3110, *ΔleuB::Pj23100 tagRFP-T::aph frt*	This work
FW1459	W3110, *ΔleuB::Pj23100 tagRFP-T::frt*	This work
FW1489	W3110, *ΔleuB::Pj23100 eqFP670::aph frt*	Wu et al. (2015)
FW2095	W3110, *ΔleuB::Pj23100 eqFP670::frt*	This work
FW1464	W3110, *hupA-tagRFP-T::aph frt*	This work
FW1495	W3110, *hupA-tagRFP-T::frt*	This work
FW2455	W3110, *hupA-mKO2::aph frt*	This work
FW2495	W3110, *hupA-mKO2::frt*	This work
FW2417	*AB1157, ori1::lacOx240::hygR, ter3::tetOx240::accC1 ΔgalK::tetR-mCerulean::frt, ΔleuB::lacI-mKate2::aph frt*	This work
FW2450	AB1157, *ori1::lacOx240::hygR, ter3::tetOx240::accC1 ΔgalK::tetR-mCerulean::frt, ΔleuB::lacI-mKO2::aph frt*	This work
FW1248	W3110, *ΔminD minE::tagYFP-minD minE::aph frt*	This work
FW1393	W3110, *ΔminD minE::tagGFP2-minD minE::aph frt*	This work
BN1406	W3110, *hupA-tagBFP::frt, ΔleuB::Pj23100 tagRFP-T::aph frt*	This work
FW1480	W3110, *ΔminD minE::yfp-minD minE::aph frt*	This work
FW1462	W3110, *ΔlacZYA::rbsexo1-yfp-minD minE::aph frt*	This work
FW1463	W3110, *ΔminD minE::sacB cat*, *ΔlacZYA::rbsexo1-yfp-minD minE::aph frt*	This work
FW1503	W3110, *ΔleuB::Pj23100 tagRFP::frt, ΔlacZYA::rbsexo1-yfp-minD minE::aph frt*	This work
FW1534	W3110, *ΔlacZYA::rbsexo2-sfGFP-minD minE::aph frt*	This work
FW1537	W3110, *ΔminD minE::rbsexo2-sfGFP-minD minE::aph frt*	This work
FW1541	W3110, *ΔminD minE::rbsendo-sfgfp-minD minE::aph frt*	Wu et al. (2015)
FW1554	W3110, *ΔminD minE::rbsexo2-sfgfp-minD minE::frt*	This work
FW1559	W3110, *ΔminD minE::rbsexo2-sfgfp-minD minE::frt, ΔleuB::eqFP670::aph frt*	This work
FW1370	W3110, *ftsZ::aph frt::envA*	This work
JKD7-1	W3110, *ΔftsZ::aph*	Dai and Lutkenhaus (1991)

### Growth Conditions

For genetic engineering, *E. coli* cells were incubated in Lysogeny broth (LB) supplemented, when required, with 100 μg/ml ampicillin (Sigma-Aldrich), 50 μg/ml kanamycin (Sigma-Aldrich), or 34 μg/ml chloramphenicol (Sigma-Aldrich) for plasmid selection, or with 25 μg/ml kanamycin, 20 μg/ml chloramphenicol, or 0.2% sucrose for selection of the genomic insertions of gene cassettes. For imaging strains with fluorescent foci with LacI fusions, we grew cells in liquid M9 minimum medium (Fluka Analytical) supplemented with 2 mM MgSO_4_, 0.1 mM CaCl_2_, 0.4% glycerol (Sigma-Aldrich), and 0.01% protein hydrolysate amicase (PHA; Fluka Analytical). For imaging other strains, we grew cells either in liquid M9 minimum medium supplemented with 2 mM MgSO_4_, 0.1 mM CaCl_2_, 0.4% glucose (Sigma-Aldrich), and 0.25% PHA, or in LB medium. For imaging, overnight cultures were back diluted into the fresh medium described above to an OD (600 nm, same below) of 0.01 in falcon tubes until an OD of 0.4–0.6 for M9 medium with 0.25% PHA and LB, and OD of 0.1 for M9 medium with 0.01% PHA. The growth conditions for the FtsZ complementation assay are as described in Osawa and Erickson (2005). 0.002% arabinose was used for the induction of ectopic FtsZswTagRFP-T fusion from the plasmids in the presence of the endogenous *ftsZ*.

### Microscopy

Fluorescence imaging was carried out using Nikon Ti-E microscope with 100X CFI Apo TIRF objective with an NA of 1.49. All fluorescent probes were excited using a Nikon Intensilight, except for the photo-bleaching of lacI foci, which was excited by SpectraX LED single-spectrum light sources (Lumencor) with SpectraX filter sets (Lumencor). For imaging with Nikon Intensilight, the λ_ex_/λ_bs_/λ_em_ wavelengths of the filter cubes are as follows: SBFP2 and EBFP2 (363–391/425/435–485 nm), TagBFP (395–415/420/435–485 nm), mCerulean (426–446/455/460–500 nm), sfGFP (450–490/495/500–550 nm), mYpet and FlAsH (490–510/515/520–550 nm), TagRFP-T and mKO2 (530–560/562/570–620 nm), mCherry and mKate2 (540–580/585/592–668 nm), eqFP670 (589–625/649/655–1200 nm). For imaging with SpectraX, the excitation filters for orange and red proteins are respectively 555/25 nm (center/width, same below) and 575/22 nm. The multiband emission filters are respectively 435/26 – 510/40 – 595/40 – 705/72 nm, and 465/25 – 545/30 – 630/60 nm. The fluorescence signal was recorded by an Andor EMCCD camera (iXon Ultra 885), with an EM gain of 100. While our emission filter for the eqFP670 extends to the infrared region, eqFP670 does not fluoresce beyond 850 nm, which is well-within the detection range for most EMCCD cameras, including the one used in this study.

### Image Analysis

Analysis of fluorescent microscopy images was carried out using Matlab with our customized programs. The background intensity was subtracted for all images individually. For identification of fluorescent LacI foci or nucleoid, the images were Gaussian blurred for subtraction, and a threshold for the expected object size and intensity was applied to eliminate noise. The intensities of the identified objects were then collected for statistics. For the photobleaching data, the mean per-pixel intensity of each nucleoid was calculated independently. To compare the photostability of more than two FPs in the main figure, the total intensities of all identified objects were summed and divided by the total number of pixel for all found objects in the first image, in order to take the completely bleached objects at the later stage of the bleaching period into account. In the latter case, the sum fluorescence intensity of the FPs in individual objects was plotted only in the supplementary figures. The standard deviation values of the intensities were not shown in the main figures for the convenience of display, and are instead plotted in supplementary figures. The matlab scripts used for these measurements can be found at http://ceesdekkerlab.tudelft.nl/downloads/.

The signal-to-noise ratio (SNR) of the cytosolic FPs is calculated using SNR_cell_ = (I_cell_-I_bg_)/SD_bg_. We use the standard deviation value of the cell-free region as a measure of background noise (SD_bg_). We use the difference between the mean intensity of a cell (I_cell_) and the mean background intensity (I_bg_) as a measure of signal. The mean and SD values of the SNR calculated from all cells are used for plotting.

## Results

### A Bright Blue Fluorescent Protein for Multi-Color Imaging in Bacteria

**FIGURE 1:**
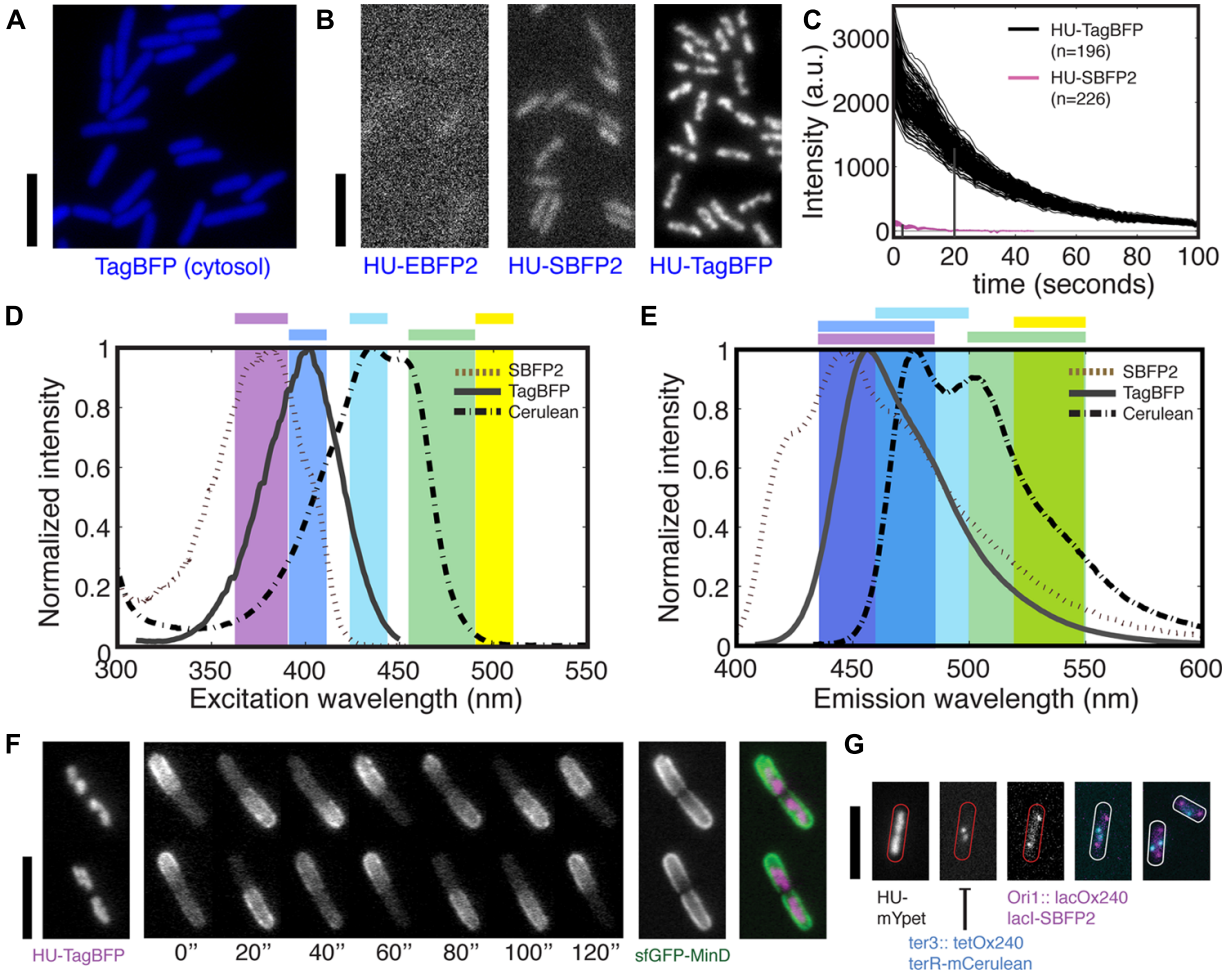
**Bright blue fluorescent proteins for multi-color imaging in bacteria. (A)** TagBFP is bright when expressed from a genomic copy under a constitutive promoter for cytosolic label in live *Escherichia coli* cell. **(B)** Fluorescent images of HU-2 tagged by three blue fluorescent protein (BFP) variants in live *E. coli* during exponential growth. **(C)** Brightness and photostability of HU-TagBFP and HU-SBFP2 in live *E. coli* cells under the constant exposure to the Nikon Intensilight with the respective filter sets. Each line represents the average intensity of one nucleoid. The index n indicates the numbers of nucleoids examined. The vertical lines indicate the bleaching halftime. **(D)** Excitation spectra of SBFP2, TagBFP, and Cerulean (a CFP), and the ranges of bandpass filters (shown in color) used for the excitation of blue, cyan, green, and yellow FPs. Note that different filters were used for SBFP2 and TagBFP due to their different excitation peak. **(E)** Emission spectra of SBFP2, TagBFP, and Cerulean, and the ranges of bandpass filters used for collecting the fluorescent light emitted by blue, cyan, green, and yellow FPs. **(F)** Endogenous fusions HU-TagBFP and sfGFP-MinD are combined for dual-color imaging. From left to right: a HU-TagBFP image, an sfGFP-MinD time series, a per-pixel standard deviation (SD) image of sfGFP-MinD calculated over time, and a false-color overlay of HU2-TagBFP and sfGFP-MinD. **(G)** Three-colors imaging of chromosome and chromosomal loci using HU2 label and two operator-repressor systems. Shown are three individual images followed by an overlay of the latter two, and an extra example. All scale bars indicate 5 μm.

The blue variants of the fluorescent proteins (BFPs) have seen few applications in bacteria due to their low brightness and short excitation wavelength (see **Table 1**). In principle, if a BFP would be sufficiently bright, it can be imaged with excitation light that is weak enough to avoid photodamage to the cells. It was recently reported that the purified monomeric mTagBFP (commercial name TagBFP) has a brightness that is similar to EGFP and 1.8 times that of EBFP2 (Subach et al., 2008). To examine its performance in bacteria, we first transformed a high-copy plasmid carrying the *tagBFP* gene under a T3 promoter into an *E. coli* strain, and indeed we observed bright blue fluorescence upon excitation through a customized filter set in a Nikon Intensilight. Furthermore, we engineered a new construct into the *leuB* locus in the *E. coli* genome, yielding strain FW1268, where the expression of *tagBFP* gene is driven by a synthetic constitutive promoter and a synthetic ribosome-binding site (RBS). The fluorescence signal of the expressed TagBFP is indeed sufficient for full cell labeling of bacteria (**Figure 1A**), indicating that TagBFP may have the brightness and maturation rate suitable for labeling proteins expressed at their endogenous level.

To compare the performance of TagBFP to other BFP variants as fluorescent tags in bacteria, we fused either *tagBFP*, *eBFP2*, or *sBFP2* genes to the 3′-end of the endogenous *hupA* gene, and examined their performance (**Figures 1B,C**). For imaging, we used two customized filter sets for the different excitation peaks of the EBFP2/SBFP2 (379 nm) and TagBFP (402 nm; **Figure 1D**), and the same emission filter (**Figure 1E**). Importantly, direct FP fusions to the C-termini of genes at their endogenous loci have been confirmed to maintain the endogenous expression level of these genes (Taniguchi et al., 2010).

HU-TagBFP outperforms HU-EBFP2 and HU-SBFP2 to a surprising extent in live bacteria, as shown in **Figure 1B**. It shows a 20 times higher fluorescence intensity than HU-SBFP2, showing clear chromosome morphologies for 200 ms exposure to half of the maximum excitation intensity provided by Nikon Intensilight. Note that the intensities of the Intensilight do vary for the different spectral range. Unexpectedly, HU-SBFP2 was threefold brighter in the stationary growth phase, despite that HU-2 was shown to be four times more abundant in the exponential phase (Ali Azam et al., 1999). Such an increase in brightness in the stationary phase was not observed in any of the other HU-2 fluorescent fusions. We hypothesize that the increase in brightness is due to a slower turnover of HU-SBFP2 proteins at stationary phase, allowing the proteins to successfully mature and fluoresce. Next to their higher brightness, the HU-TagBFP also shows superior photostability. The above constructs were used to compare the photostability of HU-TagBFP and HU-SBFP2 under the constant exposure to the same excitation light as imaged for **Figure 1B**. HU-TagBFP showed a bleaching half-time of 18 s, in contrast to the 6-s half-time for HU-SBFP2, see **Figure 1C**. In other words, the fluorescence signal of HU-TagBFP would only drop 50% after acquiring 90 images with the settings for **Figure 1B**. The initial intensity of the 196 nucleoids is measured to equal 2671 ± 354 (mean ± SD, a.u.), i.e., the HU-TagBFP concentration has a standard deviation that is only 13% of the mean value across the cell population during exponential growth in LB.

TagBFP’s excitation/emission spectra are well-separable from green and yellow fluorescent proteins (GFPs and YFPs) for multi-color imaging (**Figures 1D,E**). Taking advantage of the narrow excitation profile of TagBFP and its excitation peak at 402 nm, we customized a filter set to maximize the excitation efficiency and to collect the majority of the emitted light (**Figures 1D,E**). This filter set can be combined with regular commercial filter sets for GFP or YFP for two-colors imaging. Here, we illustrate such a combination by transducing the endogenous *hupA-tagBFP* fusion construct into a strain with a bright endogenous *sfGFP-minD* fusion (for details see **Figure 4**), yielding strain FW1561. Shown in **Figure 1F**, these two fluorescent probes successfully captured both the localizations of the nucleoids and the MinD oscillations, which together define the mid-cell for the localization of the cytokinesis machinery.

A potential combination of BFPs with cyan fluorescent proteins (CFPs) can further increase the options for multi-color imaging. Despite the higher brightness of TagBFP compared to SBFP2, it has more spectral overlap with CFP and thus is found less suitable for this application. As shown in **Figures 1D,E**, SBFP2 can be well-separated from common CFPs through customized filters, whereas the overlap between TagBFP and the CFPs is larger. Note that our current emission filter range for SBFP2 can be further adjusted to a 410–460 nm range to avoid crosstalk with CFPs. The combination of SBFP2 and SCFP3A was previously shown in Hela cells, where SBFP2 was highly expressed in the cytosol (Kremers et al., 2007). However, the limited subsequent usage in bacteria and our not fully satisfactory results for the HU-SBFP2 fusion lowered our expectations for the extent of its applications. Nevertheless, we suggest that, SBFP2 may find its application in protein co-localization studies if the morphology is not overly complicated. Here, for example, we replaced the mCherry in a LacI fusion (strain RRL189 Reyes-Lamothe et al., 2008), resulting in a LacI-SBFP2 fusion that targets the 240 *lacO* repeats in the origin region of the *E. coli* genome (strain FW1965). In this strain, the expression of the LacI-SBFP2 is constitutive. We found that it can be co-imaged with *ter* foci label TetR-mCerulean (cyan) and chromosome label HupA-mYpet (yellow; strain FW2480) (**Figure 1G**). Notably, the LacI-SBFP2 foci are much dimmer than the original LacI-mCherry fusion, requiring 2-s exposure time with Nikon Intensilight, thus less suitable for time-lapse imaging in dynamics studies. Alternatively, Everogen has produced TagCFP (Ex/Em = 457/480 nm) which can be combined with TagBFP, but we did not persue this route due to its potential spectral crosstalk with YFPs, and that our FtsZ-TagCFP fusion did not fluoresce.

### Bright and Photostable Fluorescent Proteins in the Orange-Red, Far-Red, and Near-Infrared Spectral Range

**FIGURE 2:**
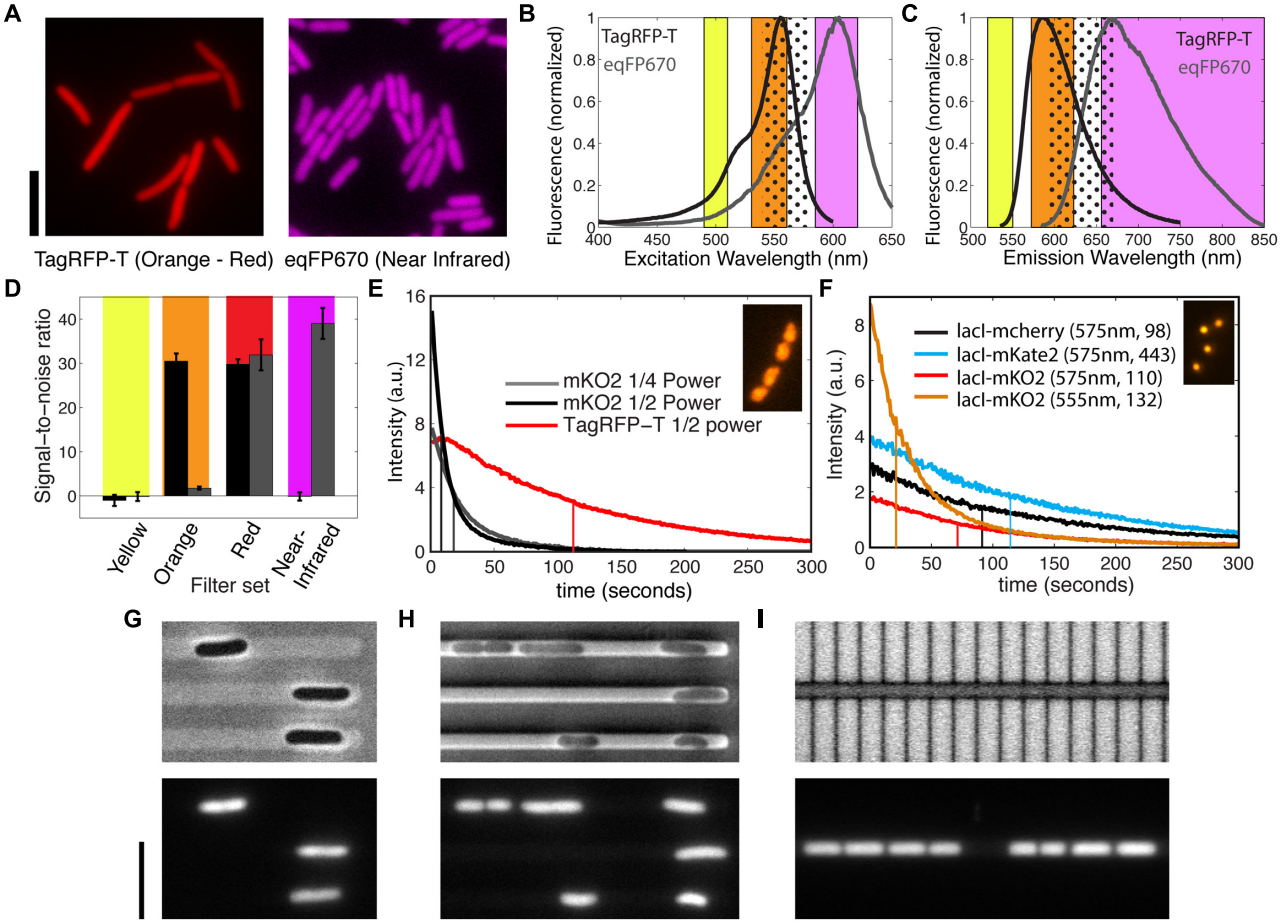
**Bright and photostable fluorescent proteins in the orange-red, far-red, and near-infrared spectral range. (A)** *E. coli* showing the fluorescence of cytosolic TagRFP-T (using orange filter set) and cytosolic eqFP670 (using near-infrared filter set) when expressed from a genomic copy under a constitutive promoter for cell body label in live *E. coli*. Scale bars, 5 μm. **(B,C)** Excitation and emission spectra of TagRFP-T and eqFP670, and the ranges of bandpass filters for the excitation and emission at yellow, orange, far-red, and near-infrared spectral range. These spectral ranges are as specified in the methods section for mYpet, TagRFP-T, mCherry, and eqFP670. The dotted regions indicate far-red filters. **(D)** Signal-to-noise ratios of TagRFP-T (black bar) and eqFP670 (gray bar) expressed in the strains shown in **(A)**, when imaged with different filter cubes shown in **(B,C)**. Here, the noise is defined by the standard deviation of the background intensity, and the signal is the difference between the fluorescence intensity of the cell and the mean background intensity. **(E)** Photobleaching of HU-mKO2 and HU-TagRFP-T in live *E. coli* cells under the constant exposure to the Nikon Intensilight at different power as indicated with the orange filter set. Inset shows a false-color image of nucleoids labeled by HU-TagRFP-T. Vertical lines indicate the bleaching halftime. **(F)** Photobleaching of chromosomal ori foci (Ori1::lacOx240) labeled by LacI-mcherry, LacI-mKate2, and LacI-mKO2 under the constant exposure to the SpectraX LED light source (1/4 power) with far-red or orange filter sets. The intensity values are the mean intensities of all the loci, i.e., the total intensity of detected loci divided by the initial number of detected loci. The indicated numbers for each probe are, respectively, the excitation wavelength and the number of spots measured. The inset shows a fluorescence image of the lacI-mKO2 foci. Vertical lines indicate the bleaching halftime. **(G–I)** Effect of microstructures made from different materials on the single-cell identifications in bright-field imaging (top panels) and using fluorescent microscopy of cytosolic labels (bottom panels). **(G)** *E. coli* cells in agarose-based microstructures, as described in Takeuchi et al. (2005). **(H)** *E. coli* cells between PDMS structures and an agarose pad, as described in Wu et al. (2015). **(I)** *E. coli* cells between silicon structures and PDMS, as described in Männik et al. (2012). The *E. coli* cells in **(G–I)** are strain BN1590 and fluorescent imaging was done in the near-infrared channel. The bright-field images in **(G,H)** were obtained through phase-contrast microscopy, whereas in **(I)** it was a regular wide-field image obtained through reflective light. Scale bar is 5 μm.

By applying the same procedure above to characterize the performance of TagRFP-T and eqFP670 (commercial name NirFP) as cytosolic labels, we found that they perform superiorly in brightness and photostability for the orange and near-infrared spectral range, respectively (**Figure 2A**). Despite the fact that these two proteins are excited with long-wavelength and hence less phototoxic compared to GFP, frequent time-lapse imaging of strains FW1459 and FW2095, with the same setting for **Figure 2A** at a frame rate of 5 s for 5 min did lead to cell growth arrest although no significant photobleaching was observed (data not shown), indicating that the photostability is not a limiting factor for imaging live cells using these FPs. Note that this is an overexposure test for whether the photostability is sufficiently high when imaging is carried out at a lower rate where photodamage is avoided. The trade-off between photodamage, fluorescence signal, and temporal resolution requires moderation of the settings to specific cases.

Notably, despite the fact that the near-infrared dimer eqFP670 was reported to have a brightness that is only 8% of EGFP as well as a modest maturation speed (Shcherbo et al., 2010), we were able to visualize the cells using either a regular far-red filter set or a customized near-infrared filter set (**Figures 2B,C**). This is largely owing to the almost full collection of far-red emission lights, while a smaller part of the emitted light is collected for the other proteins to avoid cross-talk. By contrast, a monomeric derivative TagRFP657 (excitation/emission peaks at 611/657 nm), which was produced for flow cytometer applications is not visible in our constructs even when placed under either the same synthetic promoter as above or a T7 promoter in a plasmid with pBR322 origin (pERB004 and pERB005). This agrees with the invisibility of TagRFP657 in a previous attempt in yeast (Lee et al., 2013). These facts thus make eqFP670 the most red-shifted protein applicable to bacteria so far, although we note that it’s dimeric property limits its application for protein fusions.

TagRFP-T and eqFP670 have the spectral properties that allow multi-color imaging at the red-shifted spectral range. While TagRFP-T has been reported as a red FP, its excitation and emission spectra are more blue shifted compared to conventional RFPs such as mCherry (Shaner et al., 2008). By comparing the spectral data of TagRFP-T and eqFP670, we propose that TagRFP-T can be imaged solely at the orange spectral range for excellent separation from GFPs/YFPs as well as from eqFP670, provided that latter is imaged at the near-infrared spectral range (**Figures 2B,C**). These customized filter sets allow us to measure the relative brightness of the two proteins in live cells (as shown in **Figure 2A**) from yellow to near-infrared spectral region (**Figure 2D**). Both TagRFP-T and eqFP670 are invisible with a YFP filter, while they show an equal brightness when imaged with a regular far-red filter used for mCherry. As expected from the spectral data, TagRFP-T does not show any bleed-through into the near-infrared spectral range. In the orange spectral range, TagRFP-T exhibits an over 10 times higher signal-to-noise ratio than the small bleed-through signal from eqFP670. The bleed-through from the eqFP670 into the orange spectral range is difficult to avoid due to the long tail of its excitation spectrum into the shorter wavelength. From these data, we conclude that the combination of TagRFP-T and eqFP670 is excellent for multi-color imaging at the orange and near-infrared spectral range.

As TagRFP-T was reported to be the most photostable FP at the red-shifted spectrum (Shaner et al., 2008), we set out to examine its performance as a fluorescent tag in live bacteria. We fused the 3′-end of *hupA* gene to *tagRFP-T* as well as to another recently reported FP gene, *mKO2*, which is expected to encode a bright, fast-folding orange/red FP (**Figure 2E**). Indeed, these two fluorescent probes show excellent brightness as an HU-2 tag in live bacteria, clearly improving the performance of orange proteins mKO and mOrange, which were previously shown to be invisible in live cells as fluorescent tags to FtsZ (Alexeeva et al., 2010). Under the same microscope and camera settings, HU-mKO2 appear to be 2.2 times as bright as TagRFP-T, but 15 times less photostable (**Figure 2E**, Supplementary Figure S1). Even when the exposure of HU-mKO2 was tuned to result in the same initial intensity as HU-TagRFP-T, it showed a 17-s bleaching half-time, in contrast to the 102 s for TagRFP-T. Thus, comparing these two proteins, mKO2 is more suitable for imaging proteins at low abundance, whereas TagRFP-T is more suitable for long-term time-lapse imaging.

Besides TagRFP-T and eqFP670, a far-red monomeric FP mKate2 was derived from the same origin. It showed more emission at the far-red spectral range and 57% higher brightness than mCherry (Shcherbo et al., 2009). Its photostability was shown to be similar to mCherry for purified proteins and 2.5-fold enhanced when expressed in yeast after codon optimization (Shcherbo et al., 2009; Lee et al., 2013). Here, we compare its brightness and photostability in bacteria by replacing the *lacI-mCherry* construct in strain RRL189 (see above for LacI-SBFP2 fusion) with *lacI-mKate2*. Shown in **Figure 2F**, the average intensity of the fluorescent foci of LacI-mKate2 is 25% enhanced with the same excitation/emission filters as compared to regular far-red proteins. The photostability of the two proteins is similar. In both cases after 300 s of exposure, 95% of all spots were still detectable through our automated software, showing that LacI-mCherry or LacI-mKate2 are both excellent candidates for operator-repressor system in time-lapse imaging. Furthermore, we engineered a LacI-mKO2 fusion based on strain RL189, and its foci are five times brighter in the orange spectral range than in the red spectral range (**Figure 2F**, Supplementary Figure S2). The photobleaching half-time of LacI-mKO2 is, however, 2.5 times shorter at the orange spectral range, likely due to the strong light absorbance.

Our comparison between all the orange-red and far-red fluorescent fusion tested here shows that TagRFP-T is indeed superior in photostability, agreeing with the quantitative data from the purified proteins and from fluorescent fusions in yeast (Shaner et al., 2008; Lee et al., 2013). Its far-red derivative mKate2 is indeed brighter than mChrrey in bacteria, but less significantly as previously reported in purified form and as fusion tag in yeast, which can be resulted from a slower maturation of mKate2 compared to mCherry.

Expanding the spectral range of fluorescence proteins for cytosolic label is also advantageous for studies using microstructures. Microstructures and microfluidics are emerging tools for studying bacterial physiology (Wang et al., 2010; Männik et al., 2012; Hol and Dekker, 2014; Wu et al., 2015). While these tools provide many advantages in manipulating the chemical and physical environment, they can impose challenges for imaging. In particular, the materials for the confining microstructures can make a great difference for visualizing cell body without fluorescence. For example, the structures made from agarose gel are transparent and allow phase contrast microscopy to visualize cell boundaries, owing to the fact that an agarose gel has lower refractive index (1.33, same as water) than bacteria (∼1.4; **Figure 2G**). By contrast, polydimethylsiloxane (PDMS), the most commonly used polymer for microfluidic applications, has a reflective index of around 1.4, which is almost identical to that of a bacterial cell. When imaging bacteria in between PDMS structures and agarose, the cell boundaries display much less contrast (**Figure 2H**). This type of interference between the microfluidic channel boundary and the bacterial boundary is even worsened for PDMS–PDMS interfaces. Furthermore, silicon structures are non-transparent, thus only allowing bright-field or DIC microscopy, in reflection rather than transmission, and bacteria in ∼micron-sized channels are difficult to distinguish (**Figure 2I**). In all these scenarios, the availability of cytosolic labels at the near-infrared (eqFP670) spectral range becomes particularly useful as a third or fourth color (**Figures 2G–I**, bottom panels).

### A Bright and Photostable FtsZswTagRFP-T Fusion for Multi-Color Imaging of Cell Division

As shown above, TagRFP-T can be spectrally well-separable from the near-infrared protein eqFP670 as well as from GFPs/YFPs. This can potentially lead to three-colors imaging at the long-wavelength regime, where phototoxicity can be reduced by avoiding near-UV excitations (such as necessary for CFPs or BFPs). Such an approach is in fact essential when frequent time-lapse imaging is required for tracking dynamic protein localizations over long time. Here, we describe the construction of new FtsZ and MinD fusions for time-lapse imaging of division site selection in *E. coli* with high temporal resolution.

**FIGURE 3:**
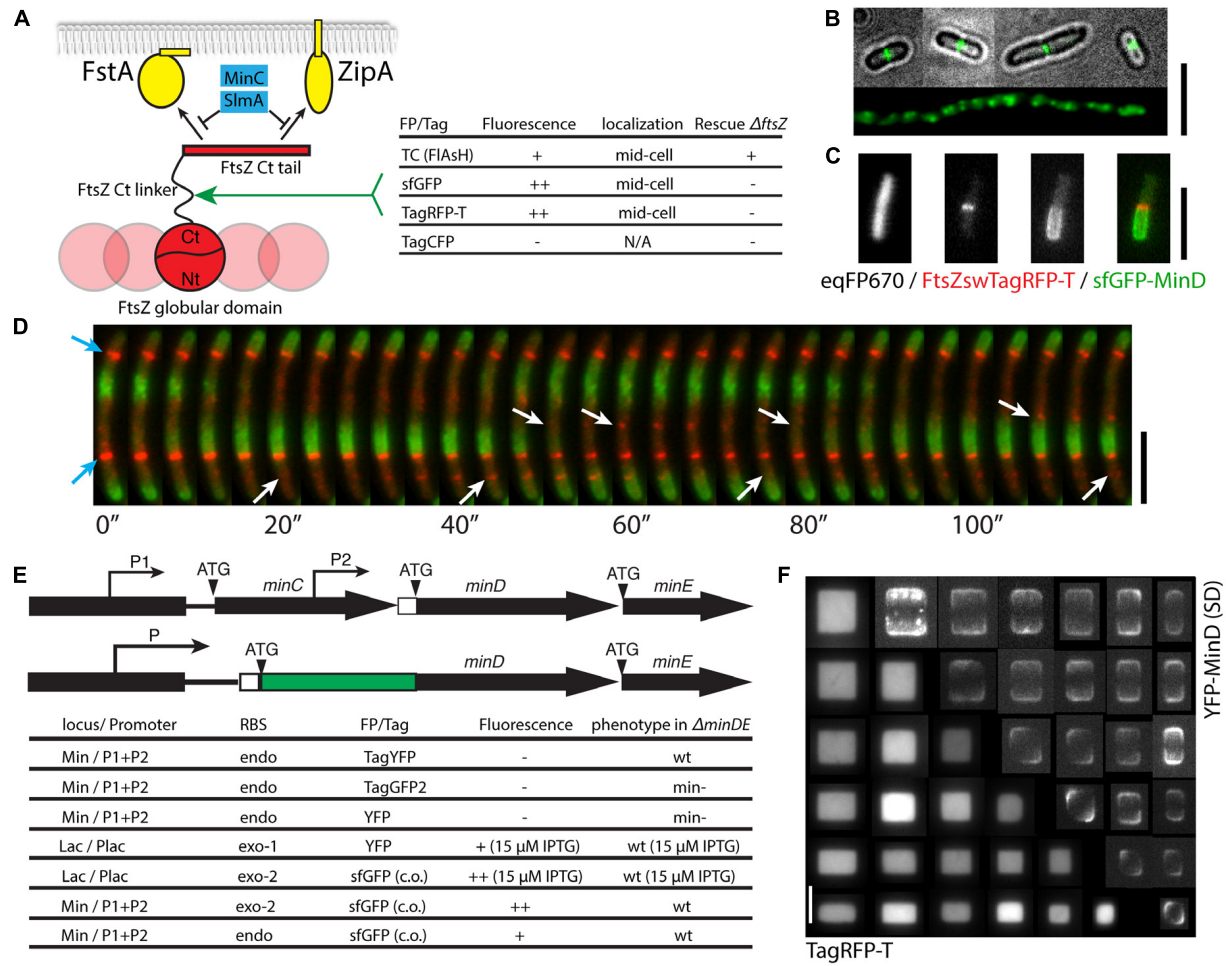
**Multi-color imaging of cell division at the green, yellow, orange, and near-infrared spectral range. (A)** A sandwich fusion strategy for labeling FtsZ and the effectiveness of using various fluorescent tags or proteins. These fluorescent probes were inserted within the flexible linker between the extreme C-terminal tail of FtsZ that is responsible for the direct interactions with FtsA, ZipA, MinC and SlmA, and the globular structure responsible for polymerization. **(B)** Fluorescence microscopy images of a strain expressing FtsZswTC labeled by the FlAsH dye (in green). The top four cells show regular rod-shaped cells (cell boundary shown in gray scale) with central Z-ring (green), while the bottom one is part of a filamentous cell in the same culture showing overexpressed FtsZ fusion proteins (green). **(C)** A combination of near infrared, orange-red and green FPs for three-colors imaging (strain FW1559 with pFWZ4, induced with 0.0002% arabinose). The FtsZswTagRFP-T was expressed from an ectopic copy under the arabinose promoter P_BAD_. **(D)** Time-lapse images showing the dynamics of FtsZswTagRFP-T and sfGFP-MinD in an elongated cell (strain FW1554 with pFWZ4, induced with 0.0002% arabinose) treated with cephalexin. Blue arrows show stable Z-rings; white arrows indicate the locations where FtsZ proteins polymerize and depolymerize. All scale bars indicate 5 μm. **(E)** Schematic and table showing the effectiveness of the fluorescent labeling of MinD at different genomic loci with different promoters and RBSs. Note that for simplicity, the RBSs of MinC and MinE are not shown. P, promoter; ATG, start codon; the white box denotes the RBS; exo, exogenous; endo, endogenous. **(F)** Fluorescent images of strain FW1503 co-expressing cytosolic TagRFP-T and YFP-MinD used in a cell shaping experiment, where single cells grow into the shapes of nanofabricated chambers, as described in Wu et al. (2015). This shows that orange-red and yellow FPs can be combined for multi-color imaging. The bottom left show cytosolic eqFP670 fluorescence and the top right images are YFP-MinD standard-deviation image calculated from the 25 images taken in 2 min.

An FtsZ molecule has a globular structure composed of independently folded N-terminal and C-terminal domains both essential for polymerization, followed by an unstructured flexible linker, and a conserved C-terminal tail responsible for interacting with its membrane-bound partners FtsA and ZipA as well as lateral interactions between FtsZ polymers (**Figure 3A**; Oliva et al., 2004; Osawa and Erickson, 2005; Shen and Lutkenhaus, 2009; Erickson et al., 2010; Buske and Levin, 2013). Biochemical and genetic studies also indicated that the C-terminal tail is the primary target of two negative regulators of FtsZ polymerization, MinC and SlmA (Shen and Lutkenhaus, 2009; Du and Lutkenhaus, 2014). So far, none of the realized fluorescent fusions of FtsZ have been shown to be fully functional, indicating that both the C-terminal tail and the globular domain are sensitive to spatial perturbations. Two close-to-functional examples are an EYFP-FtsZ fusion at temperatures below 28°C though with a very small fraction of cleaved FtsZ (Alexeeva et al., 2010), and an FtsZswVenus fusion (where Venus is inserted into the unstructured linker region of FtsZ) that rescued FtsZ deletion at 42°C after several rounds of passage likely involving mutations in the genome (Osawa and Erickson, 2005).

Since directed replacement and random insertion assays both indicated that the unstructured linker region of FtsZ can be perturbed to some extent (Osawa and Erickson, 2005; Buske and Levin, 2013), we set out to probe the possibilities of inserting other fluorescent tags at the amino acid 333 of FtsZ by adopting the strains and selection processes for FtsZswVenus (see Osawa and Erickson, 2005 and Materials and Methods; **Figure 3A**). We hoped that a FP derived from another origin could perform differently during the folding of the sandwich fusion, thus have potential to success in sandwich fusion. In order to confirm the suitability of the custom-designed flexible linkers and insertion site, we first inserted a tetracysteine (TC) sequence (FLNCCPGCCVEP) flanked by the short flexible linkers (GSGSGS-TC-GGSGSS) into the *ftsZ* gene, yielding pFWZ7. This TC tag can later be labeled by Fluorescein Arsenical Hairpin (FlAsH; Griffin et al., 1998). When induced with 0.2% arabinose, the construct pFWZ7 was able to both co-exist with the native FtsZ, and rescue the lethal effect of FtsZ deletion (**Figure 3B**). Under this growth condition, however, the *ΔftsZ* strain with pFWZ7 always showed a significant fraction of filamentous cells (30% in biomass). To label the FtsZswTC with the FlAsH dye, we optimized the protocol for live cell staining in *E. coli*. By incubating an exponentially growing bacterial culture in LB with 2 μM FlAsH dye for 3 h followed by a gentle wash step with EDT_2_ buffer, we were able to observe the fluorescence of Z-rings localizing at the cell middle in the regular rod-shaped cells (**Figure 3B**). By contrast, the filamentous cells show patches of fluorescence over the whole cell body, indicating that the filamentation is mainly caused by the overproduction of FtsZswTC, which disrupted the ratio between FtsA and FtsZ. It was shown previously that an overexpression of only FtsZ or only FtsA can lead to a filamentous phenotype (Dai and Lutkenhaus, 1992). In order to confirm that filamentation is caused by an incorrect protein expression level, we engineered pFWZ0 (Para::ftsZ) and transformed into the *ΔftsZ* strain, which rescued the cell growth upon induction but also showed a similar morphological heterogeneity. We were, however, unable to reduce the proportion of filamentous cells by lowering arabinose concentration, likely due to the inherent all-or-none expression pattern of P_BAD_ promoter. Because the FlAsH tag showed very weak fluorescence and poor photostability, we did not proceed further to optimize the construct for homogeneous induction or insertion into the genome.

We further replaced the TC peptide in the pFWZ7 construct with sfGFP, TagRFP-T, and TagCFP, which are derived from three different protein origins (**Figure 3A**). The FtsZswsfGFP and FtsZswTagRFP-T were able to form fluorescent Z-rings with or without the existence of native, untagged FtsZ, but they were not able to rescue the lethal effect of FtsZ deletion. The FtsZswTagCFP, on the other hand, did not fluoresce and was not investigated further. Nevertheless, the former two constructs can serve as alternative ectopic FtsZ probes in addition to the existing ones.

The FtsZswTagRFP-T is bright and photostable, and can be combined with eqFP670 and sfGFP-MinD for three-colors imaging (**Figure 3C**). The superior brightness and photostability of both FtsZswTagRFP-T and sfGFP-MinD allowed us to probe the dynamic relations between the localization of FtsZ and MinD with a frame rate of 1–4 s for 2 min. **Figure 3D** shows that FtsZ filaments can be assembled and disassembled within a time frame of 20–30 s, in a pattern which anti-correlates with sfGFP-MinD localization patterns. Besides the above-mentioned advantages, the use of FtsZswTagRFP-T also results in less phototoxicity due to its long excitation/emission wavelength, in contrast to the previous YFP-MinD/FtsZ-CFP combination (Shih et al., 2005).

### The Endogenous *minD* Fusion Reveals the Importance of Codon-Dependent Translational Read-Through for N-Terminal Fusions

Min proteins in *E. coli* oscillate between the two cell poles, forming a time-averaged concentration gradient of MinC, an FtsZ antagonist, with a minimum at the cell middle to allow Z-ring formation (**Figure 1F**). Without the Min system, *E. coli* often divide asymmetrically, producing anucleated minicells. The Min operon is composed of *minC*, *minD*, and *minE* genes which are positioned sequentially in the genome, with one promoter in front of MinC that drives the transcription of all three genes, and another promoter embedded in the *minC* gene to only drive MinD and MinE expression (de Boer et al., 1989; **Figure 3E**). Co-transcription of *minD* and *minE* ensures the ratio of their protein products to be rather constant, which is essential for the localization patterns of all Min proteins. Previously, *gfp-minDE* and *yfp-minDE* constructs were shown to be able to complement the *ΔminDE* phenotype when placed under the lac promoter in medium copy plasmids and induced with low concentrations of IPTG (Raskin and de Boer, 1999; Shih et al., 2002). These constructs were widely used for studying the Min protein dynamics. However, it is difficult to match inducible expression to native protein levels at all physiological conditions, and the lac promoter is known to show inhomogeneity and leaky expression.

We set out to engineer a strain where the expression of a MinD fusion will be driven by the endogenous promoter and RBS such that the expression level of the fusion will most closely reflect the native status (**Figure 3E**; Wu et al., 2015). We first constructed a few N-terminal *minD* fusion constructs that are followed by the *minE* gene and a kanamycin resistance gene. The tags are TagYFP and TagGFP2, purchased from Evrogen for their claimed photostability and fast-folding property, and YFP, from the original construct mentioned above. Unfortunately, when inserted into the *min* locus replacing a *ΔminDE::sacB-cat* cassette, none of them resulted in a strain that showed fluorescence, and only TagYFP-MinD appeared to be functional in complementing the minicell phenotype. The non-functional endogenous *yfp-minD* fusion must be caused by an insufficient expression level rather than the functionality of the fusion proteins, since YFP-MinD fusion was proven functional and fluorescent when expressed from a plasmid. On the other hand, TagYFP-MinD appeared to be expressed and functional but do not fluoresce. We next inserted the Plac::yfp-minDE construct and a new *Plac::sfGFP-minDE* construct into the genomic *lac* operon, keeping the RBS from the original plasmids, and these cells showed YFP-MinD/sfGFP-MinD oscillations, when induced using 15 μM IPTG in both wild-type and *ΔminDE* background. The sfGFP used here is a codon-optimized version. This further confirms that the difficulty in the endogenous fusion of *minD* is caused by the translational read-through when the endogenous *minD* RBS is combined with the other FP genes.

To test whether the endogenous *minD* RBS can be functional when combined with codon-optimized version of the *sfGFP* gene, we first replaced the RBS of the *Plac::sfGFP-MinD* construct with the *minD* RBS, which successfully result in sfGFP-MinD oscillations under IPTG induction. Finally, we replaced the *ΔminDE::sacB-cat* region with the *sfGFP-minDE* construct either with only the endogenous *minD* RBS or with an exogenous RBS from the plasmid behind the endogenous *minD* RBS. Both strains appeared to completely rescue the minicell phenotype (**Figure 3E**). The sfGFP-MinD fusion under the endogenous RBS was expressed at a level almost identical to the wild-type (Wu et al., 2015).

The endogenous fusion of the *sfGFP-minD*, in combination with cytosolic eqFP670 label, allowed us to study the Min pattern formation in diverse cell shapes defined by microstructures (Wu et al., 2015). By inoculating the *E. coli* cells into microchambers, we were able to mold them into defined shapes across a large range of sizes. Studying sfGFP-MinD oscillation patterns in these cells revealed that Min proteins are able to orient their oscillations according to the symmetry and scale of the cell boundary, and scale their concentration gradients with the cell dimension within a length range of 3–6 microns (Wu et al., 2015). Here, we show that also the ectopically expressed YFP-MinD fusion (in a wild-type *minDE*^+^ background, strain FW1503) can be combined with cytosolic TagRFP-T (strain FW1503) for the same applications, yielding the same localization patterns as sfGFP-MinD (**Figure 3F**). This confirms that the effect of symmetry and scale on the Min pattern formation is intrinsic and not dependent on the FPs, and verifies that the previous studies with modest YFP-MinD levels indeed represent well the native Min protein behavior. However, strain FW1503 appeared to be more sensitive to osmotic shock due to unknown reasons and thus less suitable for quantitative studies or for producing cells larger than 4.5 μm × 4.5 μm × 1 μm using the same shaping method.

### TagBFP Directly Interacts with TagRFP-T

**FIGURE 4:**
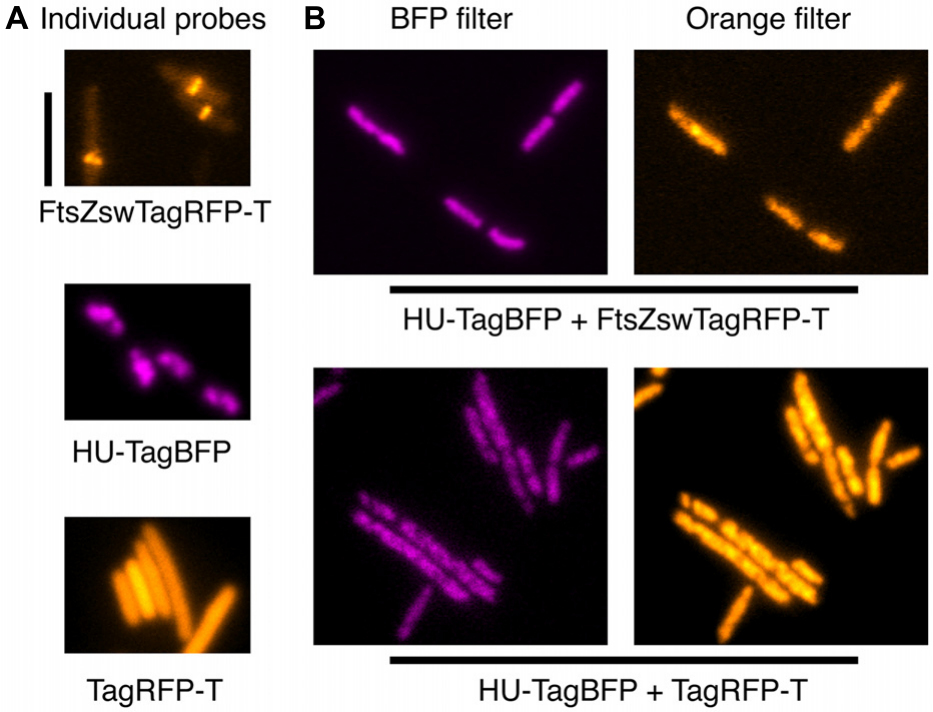
**HU-TagBFP interferes with the localizations of TagRFP-T and FtsZswTagRFP-T. (A)** False-color images of the individual fluorescent fusions. **(B)** The recruitment of both the FtsZswTagRFP-T and cytosolic TagRFP-T by the HU-TagBFP to the nucleoid, when these probes were combined. Scale bar at the top left indicates 5 μm.

From the characteristics of all FPs above, we expected that TagBFP and TagRFP-T would be excellent, mutually exclusive partners for multi-color imaging, in combination with the broadly available bright GFPs/YFPs variants. Thus, we transformed pFWZ4 (FtsZswTagRFP-T) into strain FW1359 for co-imaging of FtsZ and chromosome. To our surprise, we observed patchy blobs in the orange channel, which colocalize with the fluorescence signals from the TagBFP channel (**Figure 4**). It appears that the HU-TagBFP recruites FtsZswTagRFP-T to the nucleoid. To confirm such a strong interaction between TagBFP with TagRFP-T, we transduced a *tagRFP-T* cassette into strain FW1359, and found that HU-TagBFP is also able to recruit the freely diffusing cytosolic TagRFP-T molecules to the nucleoid (**Figure 4**). Moreover, this recruitment induced elongated cell morphology and chromosome-segregation defects (**Figure 4**).

## Discussion

We have introduced recently developed blue (TagBFP), orange (TagRFP-T and mKO2), red (mKate2), and near-infrared FPs (eqFP670) into localization studies in bacteria. These proteins generally showed the superior brightness and photostability in bacteria, consistent with previous reports of their *in vitro* behavior. This indicates that their folding properties are not the limiting factor for their general application in bacteria, contrasting earlier versions of proteins in the blue and orange spectral range, such as EBFP2 and mOrange. The near-infrared protein eqFP670 exhibited a strong brightness under our imaging conditions, presenting itself as an excellent candidate for cell body labeling for long-term and/or frequent imaging. Its extremely far-red excitation at 610 nm can minimize phototoxicity during imaging compared to all other FPs presented here. The addition of these FPs now leads to the ability to re-shuﬄe optimal choices of fluorescent probes *in vivo* for various combinations of colors. At the far-red spectral range, mKate2 showed a 25% improvement over mCherry. Since the latter is often chosen for its super-fast folding property, it is yet to be determined whether mKate2 and TagRFP-T can outperform mCherry in all aspects, such as for tagging periplasmic proteins.

For single-color imaging with high temporal resolution, TagRFP-T appears to be an excellent probe for bacterial studies. Besides its extremely strong photostability, it is excited with a much longer wavelength compared to the common GFPs. Using TagRFP-T reduces the phototoxicity compared to GFPs, allowing imaging at higher frequency and intensity. Adding to a previous study which successfully used its ancestor TagRPF as N-terminal tag (Alexeeva et al., 2010), we showed here that the brightness and photostability of TagRF-T is not perturbed by fusions to proteins either in the middle or at their C terminus, thus proving that TagRFP-T can be generally applicable in protein fusions.

For two-colors imaging, we have shown that a combination of TagRFP-T and sfGFP can allow imaging at high frequency without significant photobleaching. For three-colors protein tagging, TagBFP/GFP/mKO2 can be a good alternative to the commonly used set of CFP/YFP/mCherry. In particular, cyan FPs are mostly dimmer and less photostable than GFPs, whereas TagBFP is a fast-folding, bright and photostable variant. However, we note that the excitation spectrum of TagBFP is closer to the UV range compare to that of CFPs, which imposes a trade off between brightness and phototoxicity in time-lapse imaging. When a fourth color is required, one can add eqFP670 to the former combination (**Table 4**), whereas SBFP2 can supplement the latter. However, eqFP670 is a dimer, and thus often unsuitable for protein tagging, while SBFP2 shows a very low brightness, thus requiring either high expression levels, or strong excitation at around 380 nm.

**Table 4:** Suggestions of four-colors combinations and excitation/emission filter sets.

Four-colors				
FPs	TagBFP	sfGFP	mKO2	eqFP670
Excitation filter (nm)	395–415	450–490	530–560	589–625
Beamsplitter (nm)	420	495	562	649
Emission filter (nm)	435–485	500–550	570–620	655–1200
Detected color	Blue	Green	Orange	Near-infrared

For five-colors imaging, it is possible to combine SBFP2/mCerulean/mYPet/TagRFP-T/eqFP670 (see **Table 5**). Note that the above-mentioned limitations regarding the brightness of SBFP2 and the dimer property of eqFP670 are evident. The brightness in the blue/cyan spectrum can be improved by either engineering a bright FP that is more blue-shifted than TagBFP, or by engineering a cyan FP with a narrower excitation/emission spectrum that reduces spectral cross-talk with TagBFP. Regarding monomeric near-infrared or infrared FPs, there have been reports of newly evolved versions with an improved brightness (Yu et al., 2014). They were, however, mainly developed for deep-tissue imaging, and are yet to be tested for fluorescent tagging in bacteria for their brightness and folding properties.

**Table 5:** Suggestions of five-colors combinations and excitation/emission filter sets.

Five-colors					
FPs	SBFP2	mCerulean	mYPet	TagRFP-T	eqFP670
Excitation filter (nm)	353–391	426–446	490–510	530–560	589–625
Beamsplitter (nm)	400	455	515	562	649
Emission filter (nm)	410–460	460–500	520–550	570–620	655–1200
Detected color	Blue	Cyan	Yellow	Orange	Near-infrared

The observed direct interaction between TagBFP and TagRFP-T prohibits the combination of these two proteins in the same cell. While the dimerization or aggregation of monomeric FPs at high concentration has been recognized (Snaith et al., 2010), the possibility of two monomeric proteins interacting with each other is not commonly tested. For example, by constructing a library of individual fluorescent fusions to the same protein in yeast, Lee et al. (2013) proposed the combination of the above two proteins for multi-color imaging. Our findings emphasize that the evolution of future fluorescent probes should take the interspecies interactions into account.

Our efforts to produce HU-2, FtsZ, and MinD fusion proteins for fully replacing the endogenous ones exemplify the multi-faceted challenge regarding fluorescent tagging, which naturally depend on the structural properties of the native proteins and the transcriptional/translational read-through at their genomic loci. The fact that a short TC peptide insertion into the unstructured region of FtsZ successfully rescued the lethal effect of *ΔftsZ* but not in the case of sfGFP and TagRFP-T agrees with the finding that the unstructured region of the FtsZ has a limited tolerance to the size of a insertion (Buske and Levin, 2013). For visualizing MinD, we showed that it is possible to carry out N-terminal fusions at an endogenous genetic locus, albeit typically requiring independent sampling effort for individual genes due to the sensitivity of the transcription and translation read-through at the coding sequence close to the RBS (Kudla et al., 2009). Regarding a valid representation of a native localization pattern, a previous example showed that, while the super-fast folders sfGFP and mCherry were shown to be particularly advantageous in N-terminal or sandwich fusions and studies in periplasm (Bendezú et al., 2009; Dinh and Bernhardt, 2011; Wu et al., 2015), they were shown to form non-native foci when fused to ClpXP (Landgraf et al., 2012). Here we assessed the advantage of the recently developed FPs for studies in bacteria based on quantitative comparisons on a number of targets. We suggest that their performance as fluorescent tags be further tested in comparison to the native proteins in other future studies.

The expansion and detailed study of the FP reported in this paper will help imaging cell division in bacteria. The understanding of the processes involved in chromosome organization and cell division in bacteria has seen great development owing to the use of FPs. Current challenges in microscopy imaging of these processes lie in increasing the spatiotemporal resolution, uncovering the native behavior, and simultaneously inspecting the multiple interacting sub-components. We expect that our quantitative evaluation of novel FPs and the fusion strategies will facilitate tackling these challenges.

## Author Contributions

FW, JK, and CD conceived the experiments, FW, EVR, and BVS did the experiments, FW analyzed the data, FW and CD wrote the paper.

## Conflict of Interest Statement

The authors declare that the research was conducted in the absence of any commercial or financial relationships that could be construed as a potential conflict of interest.
