# Supplementary material for: Multi-color imaging of the bacterial nucleoid and division proteins with blue, orange, and near-infrared fluorescent proteins
Source: Front Microbiol. 2015 Jun 17;6:607. doi: 10.3389/fmicb.2015.00607 (PMC4469896; doi:10.3389/fmicb.2015.00607)
Supplement: Supplementary file 1 [file Data_Sheet_1.DOCX]

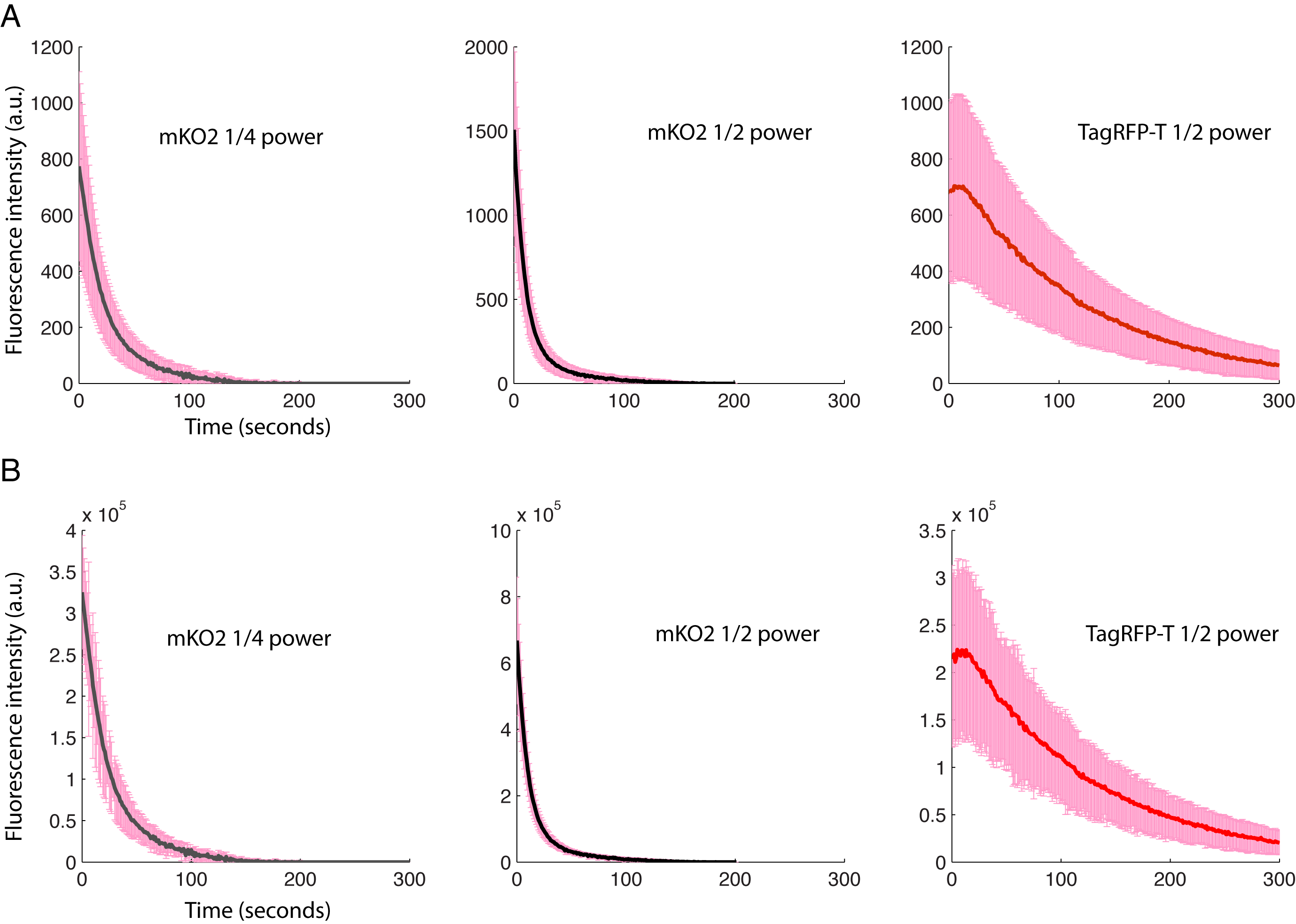


Supplementary Fig. 1. Photobleaching of orange-red fluorescent proteins when labeled to HU. A. The fluorescence intensity was calculated for all pixels in all nucleoids in the field of view. Values are the sum intensity per pixel. B. The fluorescence intensity was calculated for each nucleoid separately. Values are the sum intensity per nucleoids. The values plotted are mean and standard deviation. Note the different values along the y axes.


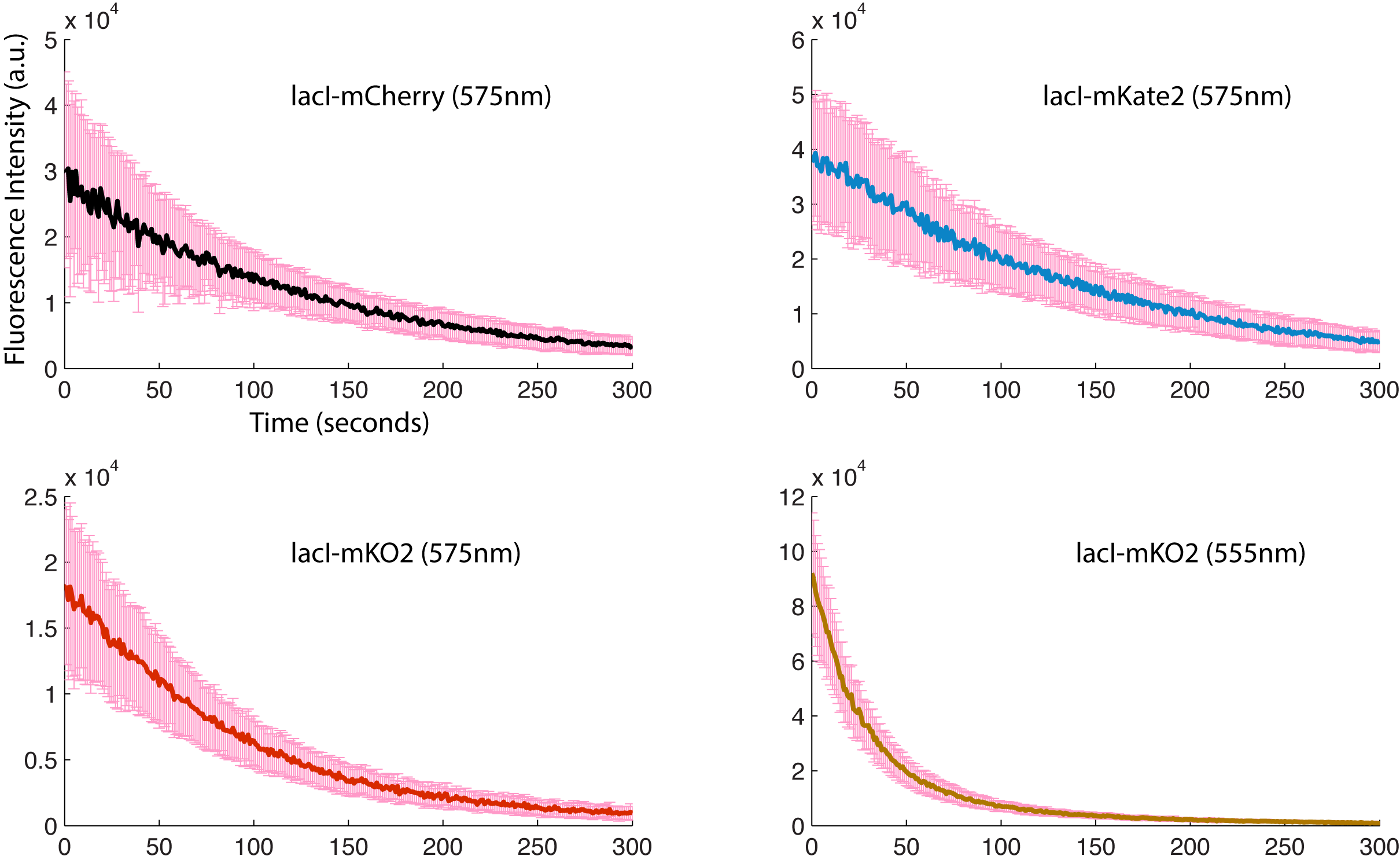


Supplementary Fig. 2. Photobleaching of orange-red fluorescent proteins when labeled to lacI. The fluorescent intensity was calculated for each detected chromosomal locus separately. The values plotted are mean intensity and standard deviation. Note the different values along the y axes.
